# Universalization of the operative strategy by systematic mesogastric excision for stomach cancer with that for total mesorectal excision and complete mesocolic excision colorectal counterparts

**DOI:** 10.1002/ags3.12048

**Published:** 2017-10-23

**Authors:** Hisashi Shinohara, Yasunori Kurahashi, Shusuke Haruta, Yoshinori Ishida, Mitsuru Sasako

**Affiliations:** ^1^ Department of Surgery Hyogo College of Medicine Nishinomiya Japan; ^2^ Department of Gastroenterological Surgery Toranomon Hospital Minato‐ku Japan

**Keywords:** D2 gastrectomy, dissectable layer, gastric cancer, mesenteric excision, stomach

## Abstract

Gastrointestinal cancer surgery aims at en bloc removal of the primary tumor with its lymphatic drainage by excising organ‐specific mesentery as an “intact package”. This concept was advocated in colorectal cancer surgery as total mesorectal excision (TME) or complete mesocolic excision (CME) procedures, but is not directly applicable to stomach cancer as a result of the morphological complexities of the gastric mesentery. In this review, we discuss the unique anatomical features of the mesogastrium by introducing its embryology, disclose its similarity to the mesosigmoid, and then propose a theoretical concept to mesentery‐based D2 gastrectomy, namely systematic mesogastric excision, which can universalize the operative strategy of stomach cancer with that of TME and CME colorectal counterparts.

## 1 INTRODUCTION

Gastric cancer remains the third leading cause of cancer‐related death worldwide.^1^ Surgery is indispensable for curing this disease; however, the extent of lymph node (LN) dissection accompanying gastrectomy has been debated for several decades. D2 lymphadenectomy, which entails systematic dissection of all the nodes along the celiac axis (CA) and its named branches as well as the perigastric nodes, has long been the standard of care in Japan and Eastern countries.^2^, ^3^ Current guidelines from Europe and the USA recommend D2 node dissection,^4^, ^5^ mainly based on the 15‐year follow‐up results from the Dutch trial showing significantly better cancer‐specific survival after D2 resection.^6^ Thus, D2 node dissection has become the best recommended practice to treat curable gastric cancer. However, to date, the theoretical background of D2 gastrectomy is not yet widely understood.

Radical surgery for gastrointestinal cancer aims at en bloc removal of the primary tumor along with its lymphovascular drainage by excising organ‐specific mesenteries.^7^, ^8^ This general concept is widely accepted in colorectal cancer surgery and is achieved by total mesorectal excision (TME) first proposed by Heald et al^9^, ^10^ or complete mesocolic excision (CME) introduced for colon malignancies based on the principles of TME.^11^, ^12^ Direct application of such mesentery‐based surgery to gastric cancer surgery is, however, not possible as a result of several anatomical restrictions of the stomach. Moreover, even if possible, it can be difficult to recognize and differentiate the mesenteries of the stomach during surgery.

In the present review, based on embryological principles, we will show that D2 gastrectomy can be theorized by introducing a concept of systematic mesogastric excision (SME), which universalizes the operative strategy of stomach cancer with that of TME or CME colorectal cancer.

## 2 EMBRYOLOGY OF THE MESOGASTRIUM

**Figure 1:**
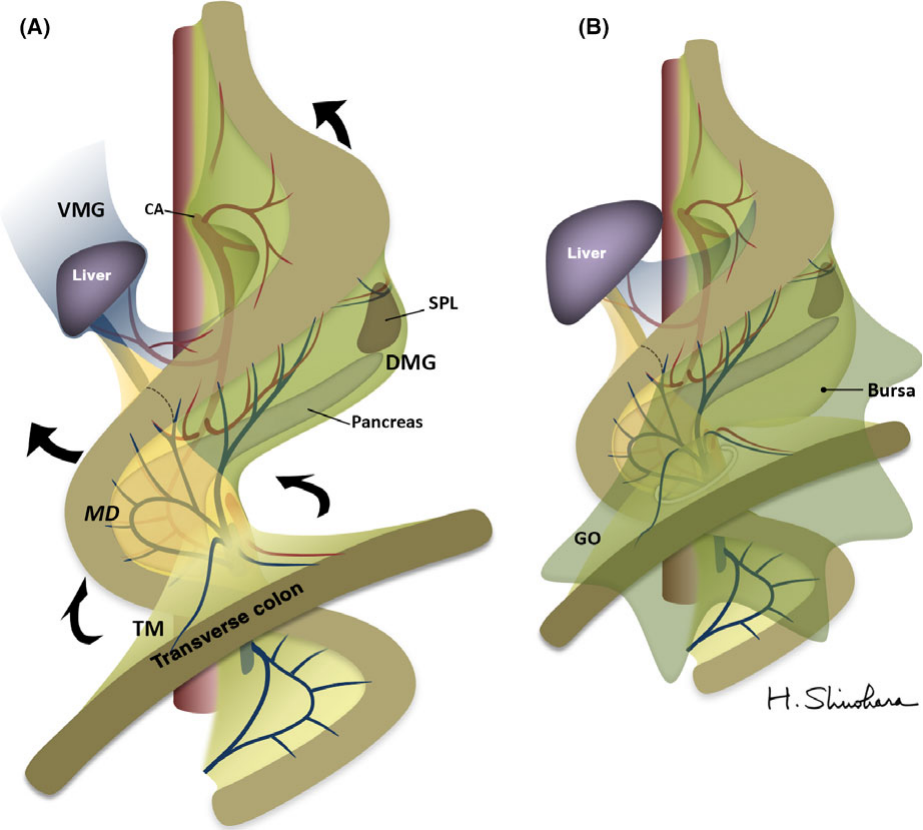
(A) Outlines of the gastric mesenteries during development. Arrows indicate concrescences of the mesenteries. (B) After development of the mesogastrium, mesoduodenum, and their derivatives. CA, celiac axis; DMG, dorsal mesogastrium; GO, greater omentum; MD, mesoduodenum; SPL, spleen, TM, transverse mesocolon; VMG, ventral mesogastrium

During the early weeks of embryological development, the primitive stomach and duodenum are suspended from the parietal wall by the dorsal and ventral mesenteries. They consist of a double layer of peritoneum that encloses pathways for vessels, nerves, and lymphatic channels by loose connective tissue spaces.^13^ Following a 90° clockwise rotation of the stomach around its longitudinal axis, the dorsal mesogastrium expands into the upper abdomen to form the omental bursa, which is then fixed to the underlying retroperitoneum (Figure 1A). The fixation is mediated by the planes in which the attachment sites of the peritoneum degenerate into thick collagen‐based connective tissue membranes.^14^, ^15^ Concurrently, the primary midgut loop rotates 270° counterclockwise around the superior mesenteric artery, bringing the proper transverse mesocolon into close proximity with the dorsal mesogastrium (Figure 1B). The mesoduodenum and transverse mesocolon is eventually overlaid with the greater omentum, an expanded derivative of the dorsal mesogastrium.^13^ The adjacent layers then fuse and account for the continuity of the transverse mesocolon and dorsal mesogastrium in the fully developed abdomen.^16^, ^17^, ^18^

The pancreas is also a mesenteric component that arises from primitive buds in the duodenal wall, grows into the mesoduodenum, and eventually extends into the dorsal mesogastrium.^13^, ^19^ In the right superior abdomen, the mesoduodenum, including the pancreas, is fixed to the dorsal parietal wall by embryological planes.^20^, ^21^ The anterior surface of the mesoduodenum is then overlaid by the proper transverse mesocolon and greater omentum, the latter derived from the expanded dorsal mesogastrium, to assume a secondary retroperitoneal position.^13^ Together, these fetal events produce certain anatomical restrictions to carrying out mesentery‐based surgery for gastric cancer.

## 3 SYSTEMATIC MESOGASTRIC EXCISION

**Figure 2:**
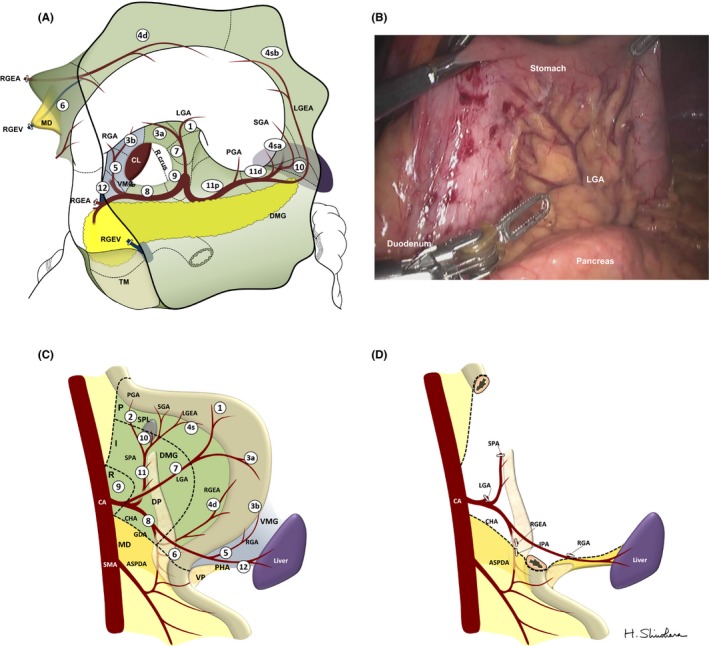
(A) Three‐dimensional overview of the mesogastrium to show mesenteric association of regional lymph stations. The omental bursa has already been opened and the right gastroepiploic artery (RGEA) and vein (RGEV) are divided. The dorsal mesogastrium (DMG) is colored green, the ventral mesogastrium (VMG) is colored blue, and the mesoduodenum (MD) is colored yellow. Numbers in circles represent regional lymph nodes. (B) Intraoperative findings of the reversed mesogastrium. (C) Image of mesogastrium with embryonic concrescences restored. The gastric mesentery can be divided into three sectors: root (R), intermediate (I), and perigastric (P) sectors. (D) Surgical concept of systematic mesogastric excision to achieve D2 lymphadenectomy by resection of the mesogastrium while sparing the pancreas and major branches of the celiac axis (CA) is shown. ASPDA, anterior superior pancreatoduodenal artery; CHA, common hepatic artery; CL, caudate lobe; DP, dorsal pancreas; GDA, gastroduodenal artery; IPA, infrapyloric artery; LGA, left gastric artery; LGEA, left gastroepiploic artery; PGA, posterior gastric artery; PHA, proper hepatic artery; RGA, right gastric artery; SGA, short gastric artery; SMA, superior mesenteric artery; SPA, splenic artery; TM, transverse mesocolon; VP, ventral pancreas

Figure 2A shows a three‐dimensional drawing of the mesogastrium in which all regional lymph stations are embedded. From the viewpoint of mesenteric structures, regional LN stations numbered by the Japan Gastric Cancer Association can be assigned to the dorsal or ventral mesogastrium. Figure 2B recreates the mesogastrium with restoration of embryonic concrescences. The dorsal mesogastrium can be divided into three sectors: root, intermediate, and perigastric sectors. Station no. 9 is equivalent to the root sector. The intermediate sector, which envelopes the pancreas, includes nodes along the left gastric artery (LGA, no. 7), common hepatic artery (CHA, no. 8), splenic hilum (no. 10), and splenic artery (SPA, no. 11). The perigastric sector includes nodes situated at the right (no. 1) and left cardia (no. 2), and lesser (no. 3a) and greater curvature (no. 4). The no. 6 infrapyloric station lies within the mesoduodenum beyond the boundary of the mesogastrium.^22^, ^23^, ^24^ The remaining few stations; that is, nos. 3b and 5, along the right gastric artery, and 12, along the proper hepatic artery (PHA) are included in the ventral mesogastrium.^25^

Dissection of N2 nodes by “entire” mesogastric excision with central vascular ligation is not achieved by the presence of the pancreas and branches arising from the CA. Ligation of the CA in radical gastrectomy is anatomically possible as the blood supply to the liver is secured in most cases by the pancreatoduodenal arcades from the superior mesenteric artery (SMA). Appleby first reported a resection of the CA along with LN for advanced gastric cancer.^26^ However, by preserving the gastroduodenal artery, even Appleby's operation cannot realize CME of the stomach.^27^ Further, as a result of severe postoperative ischemic damage of the hepatobiliary organs, the indications for Appleby's procedure remain rather limited.^28^ More importantly, the division of the CA entails combined splenopancreatectomy even when the organs are not directly invaded. Consequently, as a result of the indispensability of the major branches of the CA, “complete” excision of the mesogastrium like CME in colon cancer surgery is not feasible in gastric cancer surgery. Therefore, as shown in Figure 2C, D2 gastric cancer surgery can be achieved by en bloc excision of the mesogastrium while sparing the pancreas and its associated vessels; that is, SME.

## 4 DISSECTABLE LAYERS

**Figure 3:**
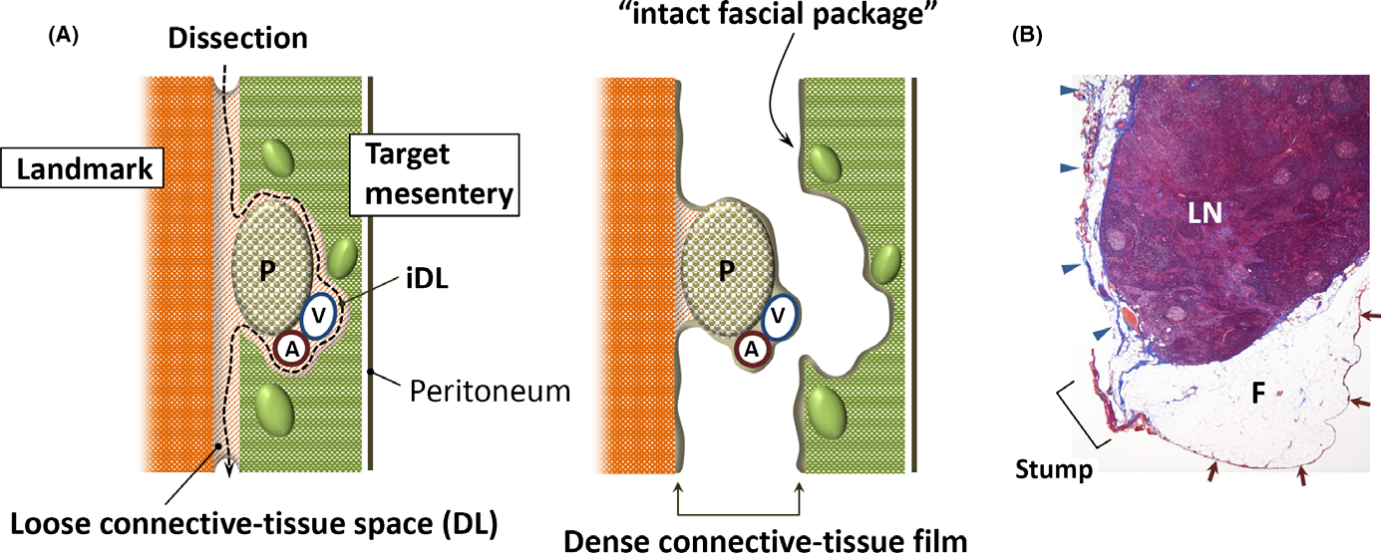
(A) Logical basis for carrying out systematic excision of the mesogastrium. Target mesentery is adherent to the landmark organs through loose connective‐tissue spaces providing surgically dissectable layers (DL). Similar areolar spaces named intramesenteric DL (iDL) exist inside the mesentery surrounding the mesenteric components, such as arteries (A), veins (V), and the pancreas (P). By sharp dissection of the DL, the target mesentery is mobilized with an “intact fascial package”. (B) Histological cross‐section (Masson's stain) of soft tissue attached with a surgical specimen. The circumferential margin is covered in a bilayered way not only on the peritoneal (arrows) but also on the detachment side by a dense connective tissue film (arrowheads), which is stained blue. F, fat tissue; LN, lymph node

The most essential process in the SME concept is to follow the areolar spaces around mesenteries. As shown in Figure 3A, the mesogastrium is adherent to the parietal wall or adjacent mesenteries through loose connective tissue spaces,^13^ which provide desired surgically dissectable layers (DL) to mobilize the target mesentery, equivalent to the “holy plane” in the TME concept.^29^ Similar areolar spaces, namely the intramesenteric DL (iDL), exist inside the mesentery surrounding the mesenteric components, such as arteries, veins and the pancreas.^22^, ^23^, ^24^, ^30^, ^31^, ^32^ Sharp dissection of the DL allows removal of the target mesentery with an “intact fascial package” while also leaving the preserved landmark organs protected by fascial coverage, because the separated connective tissue will eventually spread and attach to the detachment surface of both sides to form a dense connective tissue film. Figure 3B shows the histological findings of a cross‐section of soft tissue attached to a surgical specimen. The circumferential margin is covered in a bilayered way not only on the peritoneal side but also on the detachment side. Thus, tracing the iDL is essential to remove only the LN‐containing target mesentery while sparing the pancreas and associated vessels.

## 5 MESENTERIZATION

**Figure 4:**
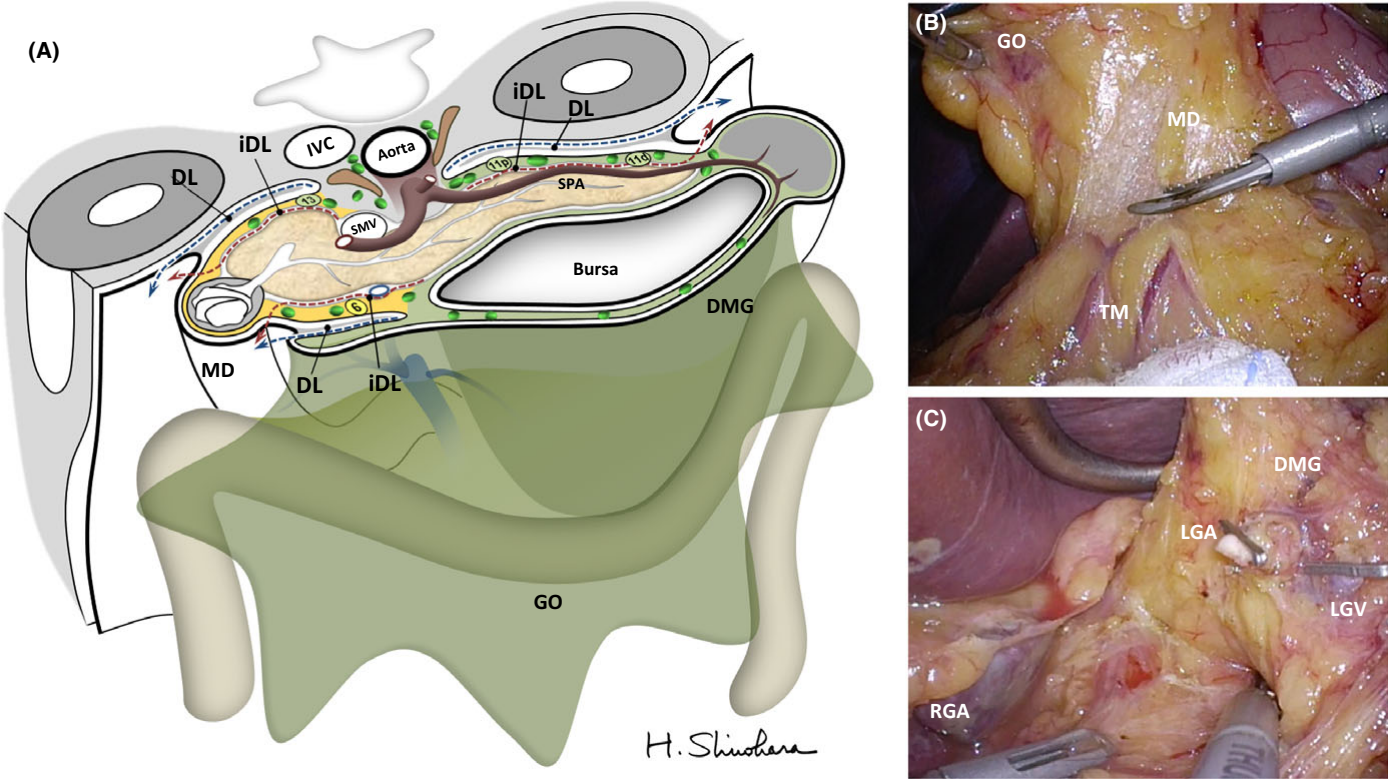
(A) Illustrated tomography of the mesogastrium indicating theoretical steps for systematic mesogastric excision: release of the mesogastric fixation from the parietal wall or adjacent mesenteries by dissociating embryological dissectable layers (DL, dashed blue arrows), followed by preservation of the pancreas and major branches of the celiac trunk by tracing intramesenteric DL (iDL, dashed red arrows). IVC, inferior vena cava; SMV, superior mesenteric vein; SPA, splenic artery. (B,C) Representative intraoperative findings after mesenterization of the mesoduodenum (MD) including the (B) no. 6 nodes, and the (C) dorsal mesogastrium (DMG) including node nos. 7, 8, 9, 11p and 12a. GO, greater omentum; IVC, inferior vena cava; LGA, left gastric artery; LGV, left gastric vein; RGA, right gastric artery; SMA, superior mesenteric artery; SPA, splenic artery, TM; transverse mesocolon

Lymphatic drainage from the stomach passes through the mesogastrium to the central nodes alongside the CA.^16^, ^33^ Figure 4 illustrates a tomography of the mesogastrium at the CA level. For isolating the mesentery, it is necessary to dissociate embryological concrescence planes. We call this procedure “mesenterization”. The loose connective tissue space is the target layer for sharp dissection. As a result of the deformation of the mesogastrium during development, such embryological planes may exist all over the stomach; for example, between: (i) the mesogastrium and left parietal wall;^14^, ^15^ (ii) the mesogastrium and transverse mesocolon;^16^, ^17^, ^18^ (iii) the mesoduodenum and right parietal wall;^20^, ^21^ and (iv) the mesoduodenum and greater omentum derived from the dorsal mesogastrium.^20^, ^30^ Figure 4B shows an intraoperative finding of the anterior surface of the mesoduodenum including no. 6 LN dissociated from the transverse mesocolon and the greater omentum. Figure 4C shows isolated dorsal mesogastrium including no. 11p LN. In both procedures, sharp dissection of the loose connective tissue space allows isolation of the target mesogastrium. Thus, mesenterization followed by tracing embryological DL would be reasonable approaches to systematic lymphadenectomy.

## 6 SPARING THE PANCREAS

**Figure 5:**
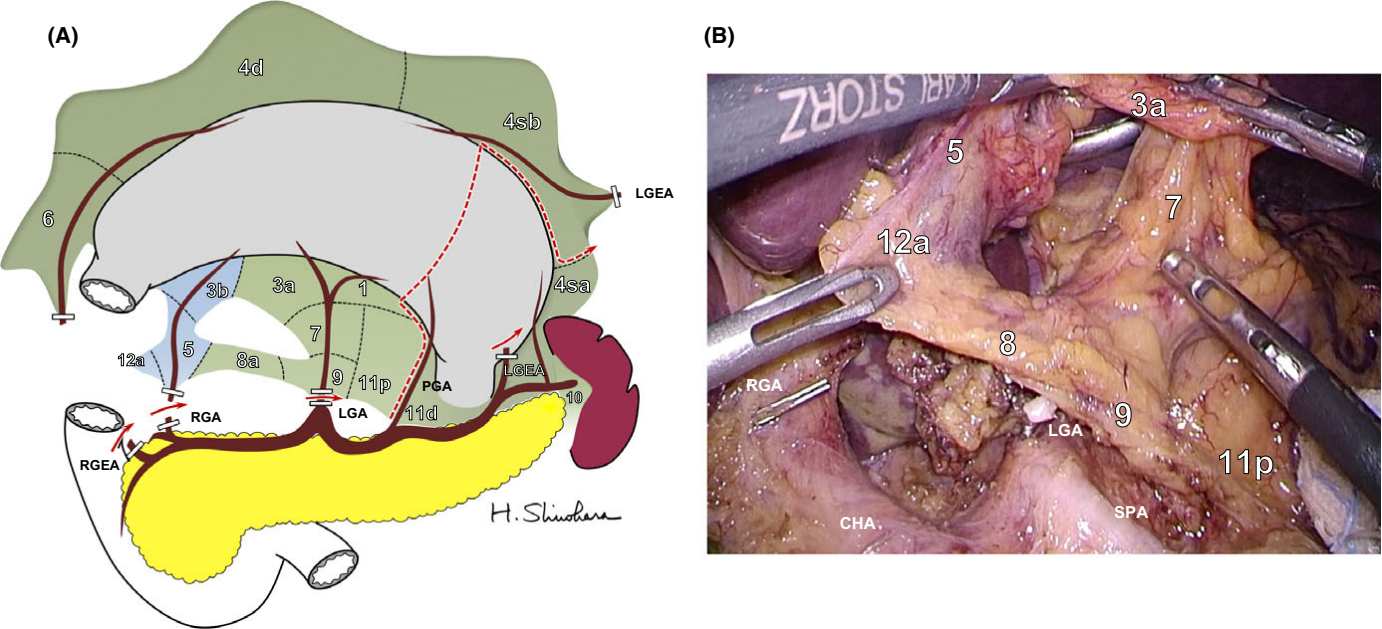
(A) Schematic illustration of excluding the pancreas and its associated vessels by tracing the intramesenteric dissectable layer with the mesogastrium. Red broken line indicates transection route of the mesogastrium when carrying out distal gastrectomy. (B) An operative finding of lymph node‐containing mesentery which was removed by tracing the intramesenteric dissectable layer along the common and proper hepatic, and splenic arteries. CHA, common hepatic artery; LGA, left gastric artery; LGEA, left gastroepiploic artery; RGA, right gastric artery; SPA, splenic artery

Totally excising the mesogastrium undisputedly results in D2 gastrectomy, but it entails combined pancreatectomy. Preservation of the pancreas also requires preservation of its associated vessels. Thus, in SME procedures, exactly tracing the iDL is another important procedure. Figure 5A illustrates an image of excluding the pancreas and major CA branches from the mesogastrium during distal gastrectomy. Under the SME concept, the arteries and veins should be ligated when they cross the iDL, but not be done after looking for their origins. Figure 5B shows operative findings of LN‐containing target mesentery which was removed by tracing iDL along the common and splenic arteries.

## 7 SURGICAL SPECIMENS OBTAINED AFTER SME

**Figure 6:**
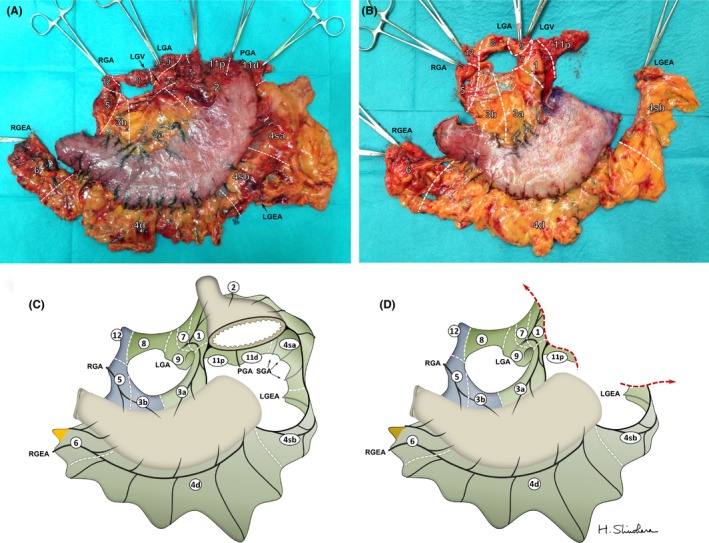
(A) Rear view of a representative specimen after total gastrectomy. (B) Frontal view of a representative specimen after distal gastrectomy. (C,D) Theoretical specimens after (A) total gastrectomy and (B) distal gastrectomy, drawn based on our systematic mesogastric excision concept. Numbers in circles represent regional lymph nodes. LGA, left gastric artery; LGEA, left gastroepiploic artery; LGV, left gastric vein; PGA, posterior gastric artery; RGA, right gastric artery; RGEA, right gastroepiploic artery; SGA, short gastric artery; SPA, splenic artery

Under the SME concept, 141 consecutive patients with stage IB or higher underwent either total (n = 40) or distal (n = 101) gastrectomy at Toranomon Hospital between October 2011 and January 2015. Figure 6A, B shows representative surgical specimens after total and distal gastrectomy, respectively, wherein the mesogastrium including all regional nodes was widely excised en bloc together with the stomach. The shape of the attached adipose tissue is comparable to the theoretical shape presented in Figure 6C, D, which were drawn based on our SME concept. In distal gastrectomy, the excision range of the mesogastrium was narrowed by omitting the dissection of the no. 2, 4sa, 10, and 11d LN. Thus, the soft tissue of specimens obtained after SME surgery formed a “mesentery” differing in size, depending on the type of gastrectomy carried out.

## 8 COMPARISON OF RETRIEVED LN NUMBERS BETWEEN D2 RESECTION AND SME

A median number of 65 (interquartile range: IQR, 48‐87) LN were retrieved from the total gastrectomy (TG) specimens. The value was significantly higher than that of 55 (range, 41‐68) from DG specimens (*P* = .022). These data were almost equivalent to those obtained by a microscopic LN retrieval method in a UK study (mean per case: 60 in total and 49 in subtotal gastrectomy)^34^ as well as to those reported in the D2 arm of the Japan Clinical Oncology Group's (JCOG) multi‐institutional randomized controlled trial 9501 (median number, 54; 102 total and 160 distal gastrectomies).^35^ LN counts in the respective LN station are shown in Table 1. The sum of each value representing almost all the LN included in the mesogastrium was calculated to be 58.5, and their distributions among the stations were almost identical to data from the D2 arm in the Dutch trial,^36^ suggesting that the SME approach does allow for adequate D2 lymphadenectomy.

**Table 1:** Lymph node counts in respective regional stations in the present study using SME compared with data from the Dutch trial

	Present series (SME)		Dutch trial (Bunt et al^36^ n = 8)
Station	n	Median (IQR)	Mean (range)
1	141	4 (2‐6)	5.5 (3‐7)
2^a^	40	2 (0‐4)	2.9 (0‐10)
3	141	8.5 (4‐14)	12.0 (3‐36)
4sa^a^	40	2 (0‐4)	12.6 (6‐21)^c^
4sb	141	2 (0‐7)	
4d	141	8 (4‐11)	
5	141	1 (0‐1)	1.0 (0‐4)
6	141	9 (5‐14)	6.0 (5‐7)
7	141	3 (1‐5)	4.0 (0‐8)
8	141	4 (2‐5)	4.0 (2‐7)
9	141	4 (2‐4)	5.3 (3‐9)
10^b^	125	5 (1‐6)	2.5 (0‐6)
11p	141	3 (1‐5)	5.7 (0‐21)^d^
11d^a^	40	2 (0‐4)	
12	141	1 (0‐1)	No data
Sum		58.5	59.8

a Removed in TG (n = 40).

b Removed in TG + splenectomy or splenopancreatectomy (n = 24).

c Expressed as the total number of lymph nodes at the no. 4 station.

d Expressed as the total number of lymph nodes at the no. 11 station.

IQR, interquartile range; SME, systematic mesogastric excision; TG, total gastrectomy.

## 9 SIMILARITIES OF THE MESOGASTRIUM AND MESOCOLON

**Figure 7:**
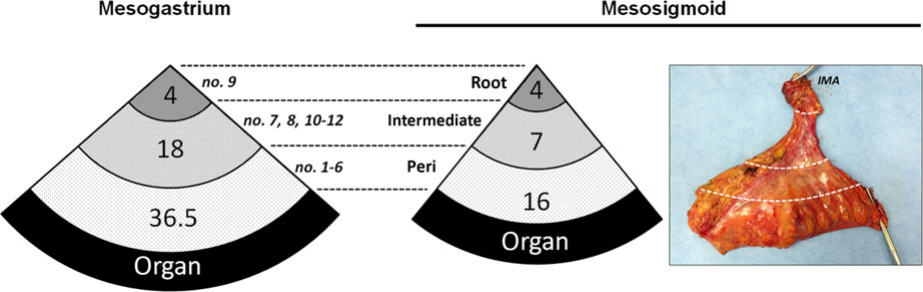
Allocations of lymph nodes in the root, intermediate and periorgan (peri) sectors of the mesogastrium and mesosigmoid. Photograph shows a representative specimen harvested after sigmoidectomy based on the complete mesocolic excision concept. IMA, inferior mesenteric artery

To compare anatomical similarities of the lymphatic stream between the mesogastrium and the mesocolon, LN counts of the mesogastrium and the mesosigmoid obtained after 157 CME procedures in the same period were compared. As shown in Figure 7, the allocation of LN in any sector decreased per the convergence of the lymphatic stream in the mesenteries, showing analogous findings between the mesogastrium (36.5, 18 and 4 in the perigastric, intermediate and root sectors, respectively) and sigmoid mesocolon (16, 7 and 4 in the pericolic, intermediate and root sectors, respectively). The slightly lower proportion of main nodes in the mesogastrium might be because of the much shorter length of the celiac trunk compared with that of the inferior mesenteric artery.

## 10 COMPATIBILITY OF SME CONCEPT IN CURRENT GUIDELINES

The current Japanese guidelines define the extent of lymphadenectomy by the type of gastrectomy.^2^ This rule is consistent with our concept as the extent of lymphadenectomy is determined by the systematic resection of the mesogastrium. In practice, we here showed that the surgical specimens obtained from total gastrectomy possess wider mesenteries, as per the theoretical shape, and contain a greater number of LN than those from distal gastrectomy. When carrying out proximal gastrectomy, only the mesentery including the left gastric and left gastroepiploic arteries should be removed.^25^ Lymphadenectomy along the infrapyloric artery may be dispensable when carrying out pylorus‐preserving gastrectomy.^24^ Thus, the extent of lymphadenectomy can be varied by systematic resection of the mesogastrium depending on the type of gastrectomy, suggesting that the background concept for lymphadenectomy in radical gastrectomy may be identical to that in colorectal cancer surgery.

## 11 SIGNIFICANCE OF SME

Metastasis to the peripancreatic N2 nodes occurs frequently; the therapeutic value of their dissection is not insignificant.^37^ Indeed, in the Dutch trial, disease‐specific survival benefit could be achieved by D2 gastrectomy.^6^ However, the early results from the Dutch trial failed to demonstrate any 5‐year survival benefits; it is noteworthy that no difference has been found in the overall 5‐year survival advantage with D2 resection.^38^ The unfavorable outcomes were expounded by significantly higher postoperative morbidity and mortality mostly associated with combined pancreatectomy carried out in 78% of patients who underwent total gastrectomy. Maruyama et al^39^ have shown that removal of the pancreas does not affect recurrence rates as lymphatic channels do not flow through the pancreatic parenchyma. Recent advances in molecular embryology have revealed that the pancreas is one of the mesenteric components arising from the duodenum under regulation of the *pdx1* and *ptf1a* genes.^19^, ^40^ Therefore, the most crucial, logical basis behind our SME concept is sparing of the pancreas from the mesogastrium to be excised. Although the pancreas was preserved in most (37 out of 40, 92.5%) patients who underwent total gastrectomy, the sum of LN and their distribution in the respective regional stations were equivalent to the data from the D2 arm in the Dutch trial.^38^

Both the omental apron and bursa, the peritoneal lining covering the pancreas and anterior plane of the transverse mesocolon are respectable portions of the mesogastrium through which lymphatic nets communicate with the stomach. Several reports have demonstrated that these regions provide implantation sites, so‐called “milky spots”, for cancer cells.^41^, ^42^ Totally excising the mesogastrium should entail omento‐bursectomy, which was considered standard in Japan from 1950’ to prevent peritoneal metastasis. However, recent results of a phase III JCOG1001 trial revealed that omento‐bursectomy provides no benefit over omentectomy alone for patients with subserosal/serosal gastric cancer.^43^ The omentum has lymphatic pathways but contains no regional LN.^41^ The current Japanese guidelines recommend omentectomy for cT3/4 gastric cancer,^2^ its significance in the overall survival rate still remains controversial.^44^, ^45^ Under the conceptual framework of SME, omentectomy is a surgical option that alters the amount of excised mesogastrium. In other words, omission of omentectomy can be interpreted as a type of reduction of the mesogastric resection. Thus, omentectomy enables wider excision of the mesogastrium, but is dispensable for D2 lymphadenectomy.

En bloc resection of the mesogastrium also seems to be beneficial for removing surgical specimens that remain intact and covered, as with an envelope. Tracing the inner DL may ensure safe exposure of the dissection margin when sparing the pancreas and its associated vessels. Etoh et al^46^ reported that extranodal metastasis, defined as the presence of cancer cells in adipose tissue, was detected in nearly 40% of patients with T4a tumors who underwent radical gastrectomy; they considered it an independent prognostic factor. Cancer cells could well spill into the peritoneal cavity from broken lymphovascular vessels during lymphadenectomy.^47^ We believe that the locoregional control offered by D2 lymphadenectomy could be improved by implementing mesentery‐based surgery.

## 12 CONCLUSION

We have proposed a surgical concept of SME which achieves D2 lymphadenectomy by en bloc resection of the mesogastrium while sparing the pancreas and its associated vessels within the mesogastrium. Under this concept, D2 gastrectomy is essentially a realization of mesentery‐based surgery despite the anatomical restrictions inherent to the mesogastrium. The concept behind SME is expected to aid the universalization of the operative strategy for gastric cancer, as have its TME and CME colorectal counterparts.

## DISCLOSURE

Funding: This work was supported in part by JSPS KAKENHI Grant Number 16H05399, and the National Cancer Center Research and Development Fund (26‐A‐4) from the Ministry of Health, Labor, and Welfare of Japan.

Conflicts of Interest: Authors declare no conflicts of interest for this article.

Approval/Consent: The institutional review board of Toranomon Hospital approved the research protocol (No. 1020). All participants provided written informed consent.
